# Antibiotic Treatment Reduces Wing Size and Alters Wing Shape in a Neotropical *Drosophila* (Diptera: Drosophilidae)

**DOI:** 10.1007/s13744-026-01442-2

**Published:** 2026-10-29

**Authors:** Marina Magalhães Moreira, Luísa de Paula Bouzada Dias, Letícia Carlesso de Paula Sena, Fernanda Mayrink Campos Pereira, Thiago Gechel Kloss, Karla Yotoko

**Affiliations:** 1 Programa de Pós-Graduação Em Entomologia, Depto de Entomologia, Univ Federal de Viçosa, Viçosa, MG Brazil; 2 Depto de Biologia Animal, Instituto de Biologia, Univ Estadual de Campinas, Campinas, SP Brazil; 3 Lab de Bioinformática E Evolução, Depto de Biologia Geral, Univ Federal de Viçosa, Viçosa, MG Brazil; 4 Lab de Ecologia E Comportamento, Depto de Biologia Geral, Univ Federal de Viçosa, Viçosa, MG Brazil

**Keywords:** *Drosophila prosaltans*, Geometric morphometrics, Gut microbiota, Tetracycline, Wing morphology, *Wolbachia*

## Abstract

Antibiotics are widely used in studies of insect–microorganism interactions, particularly to investigate symbiotic associations. However, antibiotic exposure may also induce unexpected physiological and developmental effects. Tetracycline is often used to remove *Wolbachia* from different hosts, allowing comparisons between infected and uninfected lines. This treatment, however, may also affect the host’s microbiota, physiology, and gene expression. In this study, we evaluated the effects of tetracycline on wing size and shape in 339 males of *Drosophila prosaltans* in three different generations. An original isofemale line was divided into sublines exposed to four generations of antibiotic treatment, followed by recovery generations in which the microbiota was reestablished, including reinoculation with feces from untreated males. Geometric morphometric analyses revealed a significant reduction in wing size in all treated sublines, which persisted after microbiota recovery. All sublines showed changes in wing shape after four generations of antibiotic treatment, and two returned to the original shape after recovery. These results suggest that the effects of tetracycline can persist for several generations, likely due to changes in the gut microbiota and gene expression. Such long-lasting effects may have broader phenotypic consequences and should be considered when interpreting studies that use antibiotics in host–symbiont interaction research.

**Graphical Abstract:**

Tetracycline treatment eliminated Wolbachia and reduced wing size while modifying wing shape, effects that were not fully reversed after microbiota recovery. Morphological responses varied among sublines, indicating that antibiotic effects persist even after bacterial recolonization.

**Figure:**
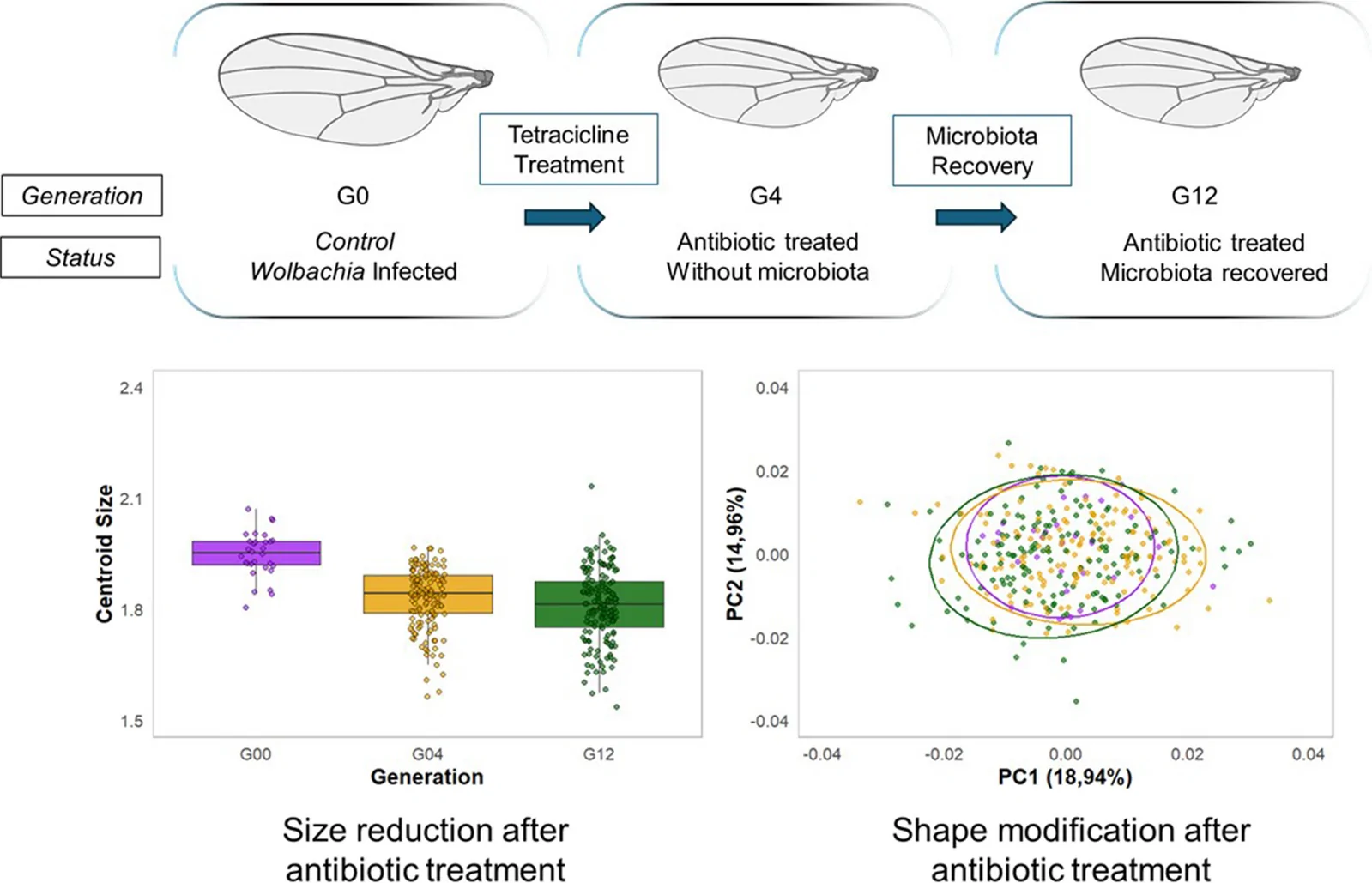

**Supplementary Information:**

The online version contains supplementary material available at https://doi.org/10.1007/s13744-026-01442-2.

## Introduction

Insects are constantly exposed to antibiotics, either through the improper disposal of medications or through their use to control infectious agents in animal and plant production (Witte 2000). Antibiotics are used to promote animal growth in agriculture (Dibner and Richards 2005), to combat bacteria that infect plants (McManus et al. 2002; Batuman et al. 2024), and to control vector-born diseases (Doudoumis et al. 2013), making them increasingly present in the environment (Kumar et al. 2005).

While antibiotics are beneficial in agriculture, scientific research, and disease treatment, they can also cause negative side effects in individuals. These effects occur because of the biochemical similarities between bacteria and eukaryotic mitochondria, particularly in their ribosomes (Zhang et al. 2005). Exposure to antibiotics can interfere with mitochondrial protein synthesis (Kalghatgi et al. 2013; Wang et al. 2015). When used prophylactically in insect rearing, antibiotics may increase juvenile mortality, alter development time, pheromone production, fatty acid, or amino acids levels, and reduce fertility (Sikorowski and Lawrence 1994; Dickel et al. 2016). In *Chrysomya albiceps* (Wiedemann) (Diptera: Calliphoridae), exposure to moderate to high antibiotic concentrations exceeded the detoxification capacity of individuals, resulting in reduced longevity (Renault et al. 2018). Antibiotics can also alter gene expression patterns, mainly affecting the immune and digestive systems of treated individuals (Zhang et al. 2023; Kriete and Scott 2025). Furthermore, they may influence the expression of factors involved in sexual recognition, making treated individuals less attractive to females (Kriete and Scott 2025).

The study of interactions between *Wolbachia* and arthropods is one of the main experimental systems that rely on antibiotic use (Li et al. 2014). *Wolbachia* belongs to the order *Rickettsiales*, and it is present in approximately 52% of arthropod species (Weinert et al. 2015). This endosymbiont occurs in several host tissues, including the gut, Malpighian tubules, fat body, muscles, brain, and reproductive organs (Pietri et al. 2016). Its primary mode of transmission is vertical, from females to their offspring, and it can induce various reproductive phenotypes that enhance the fitness of infected females (Werren et al. 2008).

The main phenotypes induced by *Wolbachia* include feminization (Werren and Beukeboom 1998; Narita et al. 2007; Negri et al. 2008), parthenogenesis (Stouthamer et al. 1990; Arakaki et al. 2001; Weeks and Breeuwer 2001), male embryo death (Dyer and Jaenike 2004; Kageyama and Traut 2004), and cytoplasmic incompatibility (Werren 1997; Beckmann et al. 2017). Because *Wolbachia* can also infect the brain, there is evidence that it interferes with the reproductive behavior of its hosts (Bi and Wang 2020). In addition, *Wolbachia* may increase the fertility of infected females (Fry et al. 2004; Weeks et al. 2007), provide protection against viral infections (Teixeira et al. 2008; Bruner-Montero and Jiggins 2023), and support the acquisition or use of limited nutrients in the environment (Newton and Rice 2020). Investigating *Wolbachia*-induced phenotypes requires the use of uninfected lines, which are most commonly established through antibiotic treatment with compounds such as tetracycline or rifampicin (Li et al. 2014).

Tetracycline is widely used to study interactions between *Wolbachia* and different species of *Drosophila* (Diptera: Drosophilidae) (Li et al. 2014). However, it can produce several negative effects on treated hosts. This antibiotic is strongly associated with reduced mitochondrial efficiency and disruption of proteostasis in both vertebrates and invertebrates (Houtkooper et al. 2013). In *Drosophila simulans* (Sturtevant) (Diptera: Drosophilidae), tetracycline directly impairs mitochondrial function and decreases ATP synthesis (Ballard and Melvin 2007). Multigenerational tetracycline treatment has been shown to alter gene expression, particularly in genes linked to immune and digestive systems (Zhang et al. 2023; Kriete and Scott 2025). Additionally, Zhang et al. (2023) demonstrated that four generations of tetracycline exposure in ticks caused damage to the intestinal epithelium, thereby compromising feeding. Considering these negative effects, findings including increased fertility in infected females, improved nutrient uptake, and behavioral changes may, in fact, reflect consequences of the antibiotic treatment to create uninfected lines. Consequently, adverse outcomes observed in treated lines may not result directly from *Wolbachia* removal, but rather from the physiological effects of tetracycline, potentially leading to misinterpretation of experimental results.

Considering the potential side effects associated with antibiotic use, this study investigated whether tetracycline treatment alters wing morphology in *Drosophila prosaltans* (Duda) (Diptera: Drosophilidae), a neotropical drosophilid that harbors the *Wolbachia* strain *w*Pro (Miller and Riegler 2006). Because wings play a key role in mating and courtship, male wings were analyzed as a proxy for potential fitness-related consequences of the treatment (Partridge et al. 1987; Menezes et al. 2013). Moreover, given that gut microbiota influences multiple aspects of host development and physiology (Sharon et al. 2010; Engl et al. 2018; Chiang et al. 2022), the natural microbiota was reintroduced to treated individuals using feces from untreated males in an attempt to restore the natural microbiota, allowing us to distinguish microbiota-mediated effects on wing morphology from the direct or residual effects of tetracycline. Therefore, this work isolated the effects of tetracycline on wing morphology, independent of microbiota influences, providing a controlled framework to evaluate the consequences of antibiotic exposure across generations.

## Materials and Methods

### Maintenance of Laboratory Lines

To evaluate the effects of antibiotic treatment on wing shape, we used the *D. prosaltans* isofemale line Pro 101. This line was collected in 2016 from an urban fragment of Atlantic Forest located on the campus of the Federal University of Viçosa (20°45′48″S; 42°51′53″W) and had been maintained under laboratory conditions for approximately 80 generations. Since its collection, the line has been reared in 2.5-cm diameter test tubes containing a banana–barley culture medium (Moreira et al. 2026 – Supporting Information 1). The flies are maintained at 23 °C under a 12L:12D photoperiod in a Biochemical Oxygen Demand (BOD) incubator. Periodic checks are performed to remove contaminants such as mites and fungi from the tubes, and new tubes are established using newly emerged adults.

### Detection of *Wolbachia*

#### DNA Extraction

Genomic DNA was extracted from pools of five individuals from each generation of each subline. Pooling individuals increased the probability of detecting *Wolbachia* infection within the evaluated sublines. Extractions were performed using the Wizard® Genomic DNA Purification Kit (Promega A1120), following the manufacturer’s instructions with minor volume adjustments as described in Moreira et al. (2026). Samples were macerated in EDTA/Nuclei Lysis Solution, subjected to Proteinase K digestion at 37 °C, and proteins were precipitated using Protein Precipitation Solution. DNA was precipitated with isopropanol, washed with 70% ethanol, rehydrated in DNA Rehydration Solution, treated with RNase, and stored at −20 °C until further analyses.

#### PCR Reactions to Detect *Wolbachia* Infection

To assess DNA integrity, samples were quantified and subjected to standard PCR amplification of the mitochondrial COI gene using primers CO1 106 F (5′ ATTCAGAATATCTATGTTCAG 3′) and CO1 154R (5′ TTTAATTTTACCTGGATTTGG 3′) (Madi-Ravazzi et al. 2021). PCR reactions were performed in a final volume of 10 µL containing 1× GoTaq Green Buffer (Promega, Madison, USA), MgCl_2_ (3 mM), dNTPs (0.04 mM), primers (0.4 µM each), template DNA (final concentration of 5 ng/µL), 0.1 µL GoTaq G2 DNA Polymerase (Promega, Madison, USA), and ultrapure water to complete the reaction volume. Thermal cycling consisted of an initial denaturation at 94 °C for 5 min, followed by 30 cycles of 94 °C for 30 s, 55 °C for 30 s, and 72 °C for 1 min, with a final extension at 72 °C for 5 min. Samples that failed to amplify were excluded from subsequent analyses.

The *Wolbachia* infection status of the lines was determined using standard PCR targeting the *wsp* gene (*Wolbachia* Surface Protein) with primers wsp-226F (5′-GTGGTGGTGCATTTGGTTATAAAATGG-3′) and wsp-588R (5′-CATAAGAACCGAAATAACGAGCTCCAG-3′) (Arthofer et al. 2009). PCR reactions were performed in a final volume of 10 µL containing 1× GoTaq Green Buffer (Promega, Madison, USA), dNTPs (0.04 mM), primers (0.3 µM each), template DNA (final concentration of 5 ng/µL), 0.1 µL GoTaq G2 DNA Polymerase (Promega, Madison, USA), and ultrapure water to complete the reaction volume. Thermal cycling consisted of an initial denaturation at 94 °C for 2 min, followed by 30 cycles of 94 °C for 30 s, 67 °C for 45 s, and 72 °C for 30 s, with a final extension at 72 °C for 5 min. PCR products were visualized on a 1% agarose gel, and the presence of a 365 bp band was interpreted as a high-titer infection. All PCR reactions included a positive control (*Drosophila sturtevanti* (Duda) (Diptera: Drosophilidae) – st 22, infected with *Wolbachia*), a negative control (*D. sturtevanti* – st 18, uninfected), an extraction blank (tube without tissue), and a PCR blank (tube without DNA) to prevent misinterpretation due to contamination.

### Antibiotic Treatment to Eliminate *Wolbachia*

**Fig. 1:**
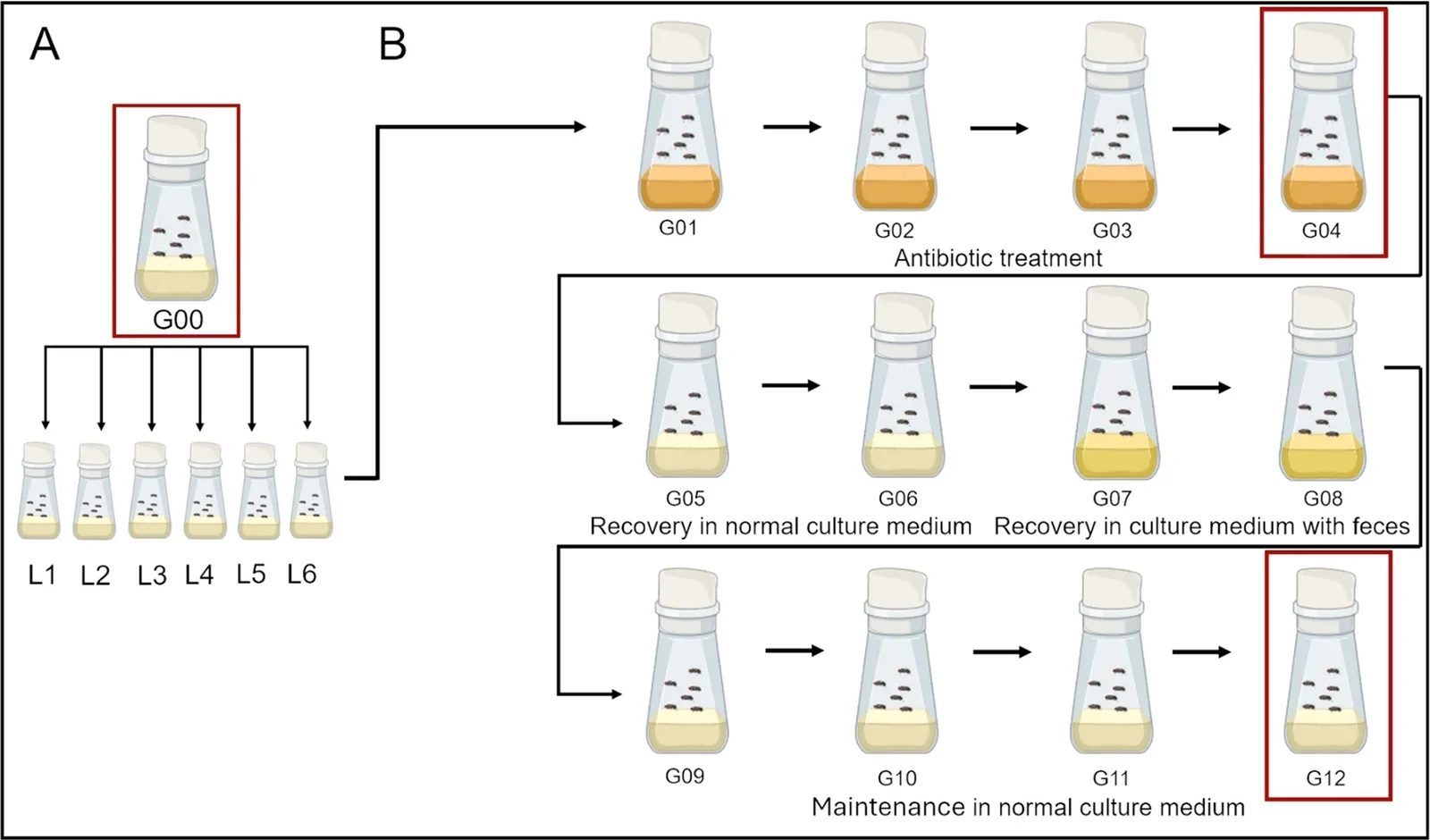
Schematic representation of antibiotic treatment in an isofemale line of *Drosophila prosaltans* (Pro 101 – G00) to generate sublines with *Wolbachia* levels undetectable by PCR. (**A**) Individuals from the infected G00 line were divided into six sublines for antibiotic treatment. (**B**) Each subline underwent four generations in medium supplemented with 0.03% tetracycline (G01–G04), followed by two generations in standard medium without antibiotics (G05–G06), and two generations in medium supplemented with feces from males of the original line to restore microbiota (G07–G08). Sublines were then maintained for four additional generations in standard medium without antibiotics or fecal supplementation (G09–G12). Red rectangles indicate the three generations evaluated in the analyses. Illustration created with BioRender.com

To evaluate the effects of tetracycline on wing size and shape in *D. prosaltans*, the original Pro 101 line was divided into six sublines, which were treated independently (Fig. 1A). Because all sublines share the same genetic background, this design allowed us to assess whether treatment effects occurred consistently across all of them. Sublines were maintained in a BOD incubator at 23 °C under a 12L:12D photoperiod. They were checked weekly to prevent generational overlap by transferring newly emerged adults to new vials.

The treatment was carried out by adding tetracycline to culture medium at a final concentration of 0.03% in 6.0 cm diameter vials. This concentration has been shown to reduce *Wolbachia* titers in *Drosophila* species to levels undetectable by PCR (Reynolds et al. 2003; Caragata et al. 2013; Strunov et al. 2013; Li et al. 2014). Previous studies and treatment protocols commonly used at least three generations of antibiotic exposure for *Wolbachia* removal, with additional generations applied when necessary to ensure consistent elimination (Li et al. 2016; Zhang et al. 2020; Cooper and Shropshire 2024; Kriete and Scott 2025). Therefore, the sublines were maintained in antibiotic medium for four consecutive generations (Fig. 1B), where females laid eggs and larvae developed. After each generation, the *Wolbachia* infection status was assessed by PCR to verify treatment effectiveness. Adults from each generation were preserved in 99.8% ethanol for subsequent morphometric analysis.

### Recovery from Antibiotic Treatment

Given the key role of gut microbiota in nutrient absorption, behavior, physiology, hydrocarbon production, and its potential influence on individual development (Sharon et al. 2010; Engl et al. 2018; Chiang et al. 2022), we performed a microbiota recovery procedure by reintroducing the natural gut microbiota to treated individuals (Poinsot and Merçot 1997; Li et al. 2014). This step was critical to distinguish microbiota-mediated effects on wing morphology from the direct or residual effects of tetracycline and allowed the evaluation of antibiotic treatment effects in *Wolbachia*-free individuals retaining a natural microbiota.

At the end of the fourth generation of tetracycline treatment, sublines were transferred to vials with standard culture medium, without antibiotics, for two generations. They were then maintained for two additional generations in medium supplemented with feces from males of the original Pro 101 line in an attempt to restore the gut microbiota (Douglas 2018) (Fig. 1B). For the preparation of the supplemented medium for each subline, 20 males from the original Pro 101 line were placed in a vial for 48 h to allow feces deposition on the culture medium. After this period, the males were removed, and newly emerged adults from the recovering sublines were transferred to the vials containing the feces-enriched medium. This procedure was performed separately for each subline and was repeated throughout the two generations of feces supplementation. Microbiota recovery was expected to occur through feeding of both larvae and adults on the feces-supplemented medium (Weiland et al. 2022). After recovery, sublines were kept in standard culture medium. Because the bacterial community was not directly profiled, the term “restored microbiota” is used here to refer to functional recolonization, rather than an exact reconstruction of the original community composition.

Following each recovery generation and during periodic checks, PCR tests were conducted to confirm the absence of detectable *Wolbachia*. Adults from all recovery and subsequent generations were preserved in 99.8% ethanol for morphometric analyses. For downstream analyses, the G00 generation served as the control group, consisting of *Wolbachia*-infected individuals without antibiotic exposure; G04 corresponded to the antibiotic-treated generation with depleted microbiota; and G12 represented the antibiotic-treated generation with the natural microbiota restored (Fig. 1B).

### Geometric Morphometrics of Wings

**Fig. 2:**
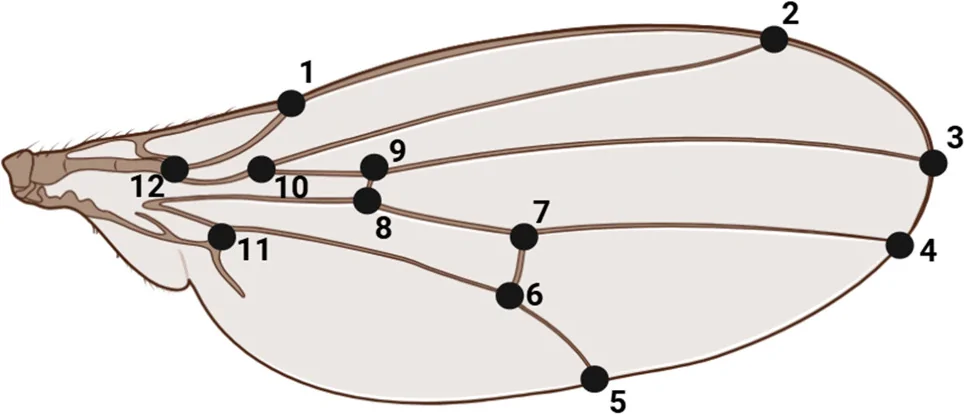
Dorsal schematic view of the right wing with anatomical landmarks of type I, based on Prestes et al. (2021). Illustration created with BioRender.com

For geometric morphometric analyses, the right wings of 339 individuals were examined. A minimum of 30 males from the initial infected line (G00 – control) and 30 males from each treated subline were dissected in G04 (antibiotic-treated with depleted microbiota) and G12 (antibiotic-treated with restored microbiota) (Fig. 1B). The exact number of individuals analyzed for each subline and generation is provided in Supporting Information 2. Wings were mounted on glass slides with a millimeter scale and photographed using a Nikon SMZ 745 T stereomicroscope equipped with a Labomed iVu 3100 camera. Images were imported into TpsDig v.2.32 (Rohlf 2015) to mark the scale and identify type 1 anatomical landmarks (points of vein intersections or intersections with the wing edge) (Fig. 2). Wings lacking all landmarks were excluded from the analyses. TpsDig converted landmark points into Cartesian coordinates, which were analyzed using MorphoJ v.1.07 (Klingenberg 2011). Each wing was digitized twice on separate days to assess the accuracy of landmark placement and avoid confounding measurement errors with true variation. In MorphoJ, Procrustes superimposition was applied to the coordinates to compare wing shapes while removing variation due to rotation, translation, and scale.

#### Wing Size Analysis

Wing size was estimated as centroid size, defined as the square root of the sum of squared distances from each anatomical landmark to the wing centroid, and computed for the right wings of males in MorphoJ v.1.07 (Klingenberg 2011). Centroid size was used as a proxy for individual body size. For each subline in each generation (G00, G04, and G12), at least 30 males originating from the same culture vials were analyzed (Supporting Information 2). The data were exported and analyzed in R (R Core Team 2025). Because the control generation consisted of a single ancestral line and only one culture vial was produced for each generation in each subline, subline could not be treated as a random effect in mixed-effects models, since no biological replication at the subline level was available. Therefore, differences in centroid size between generations were assessed using a linear model with a normal distribution, considering centroid size as the response variable and generation as the explanatory variable. After model fitting, multiple comparisons between generations were performed using Tukey’s test with the glht() function from the *multcomp* package (Hothorn et al. 2008). The same analysis was conducted separately for each subline, comparing the control group (G00) with the generation antibiotic-treated with depleted microbiota (G04) and the generation antibiotic-treated with restored microbiota (G12).

#### Wing Shape Analysis

Procrustes superimposition was performed in MorphoJ v.1.07 (Klingenberg 2011) on the right wings of males, grouped by generation (G00, G04, G12) and by subline. This procedure removes variation due to rotation, translation, and scale, ensuring that observed differences reflect only wing shape variation. Following Procrustes superimposition, allometry (the effect of size on shape) was assessed. To compare wing shape among the generations (G00, G04, and G12), allometry was corrected using a common slope pooled across groups. In comparisons within each subline across generations, allometry was corrected using group-specific slopes. When allometry was significant, residuals from the correlation between size and shape were used in subsequent analyses to isolate shape variation. Principal component (PC) values for shape were calculated in MorphoJ v.1.07 (Klingenberg 2011) and exported for further analysis.

Statistical analyses were conducted in R (R Core Team 2025) using the *geomorph* package (Baken et al. 2021; Adams et al. 2025). Because the control generation consisted of a single ancestral line and only one culture vial was produced for each subline generation, subline could not be treated as a random effect in mixed-effects models or hierarchical permutation designs. Shape differences between generations and within sublines were evaluated using a Procrustes-based multivariate linear model (procD.lm). This permutation-based approach does not rely on strict distributional assumptions and is widely considered robust for geometric morphometric datasets. Analyses were performed using principal components (PCs) explaining 95% of shape variation, reducing dimensionality while preserving most biologically meaningful variation. Pairwise differences between groups were assessed using the pairwise function. Statistical procedures were implemented via RRPP (Collyer and Adams 2018, 2024).

## Results

### Antibiotic Treatment and *Wolbachia* Detection

The four generations of treatment of the sublines with tetracycline occurred between May and October 2023, each generation lasting approximately 30 days. In the third generation of antibiotic treatment, *Wolbachia* could no longer be detected by conventional PCR in any of the six sublines. This absence was maintained through the fourth and final treatment generation, confirming the effectiveness of the antibiotic treatment.

The four generations of recovery occurred between October 2023 and February 2024. During the first recovery generation, the L5 subline was lost, as it did not produce larvae, ending in G05 (Fig. 1B). This pattern is consistent with a possible fitness cost associated with tetracycline treatment and microbiota disruption. Recovery continued in the other sublines. After the four recovery generations and in all subsequent generations, *Wolbachia* remained undetectable by conventional PCR in the pools of individuals tested from each subline. Although conventional PCR may not detect very low-titer infections, the repeated negative results across generations suggest that the antibiotic treatment was effective in substantially reducing or eliminating *Wolbachia* infection in the treated sublines.

### Variation in Centroid Size

**Fig. 3:**
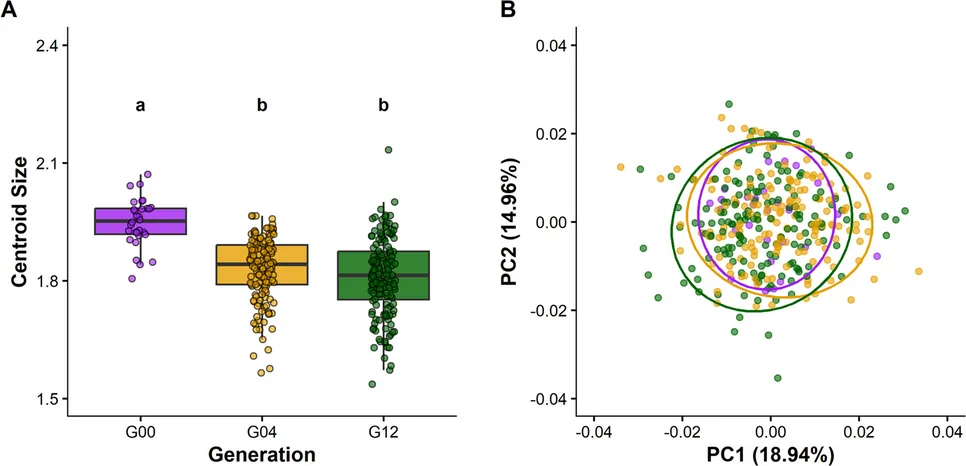
Variation in centroid size (**A**) and wing shape (**B**) among the control generation (G00) in purple, antibiotic-treated generation with depleted microbiota (G04) in yellow, and antibiotic-treated generation with restored microbiota (G12) in green. **A** Boxplot of centroid size across generations showing the interquartile range (25%–75%) and median. **B** Principal component analysis (PCA) plot based on allometry-corrected shape residuals (size effects removed), represented by the first two components (PC1 and PC2), illustrating wing shape variation among generations. Percentages indicate the proportion of shape variation explained by each component. The ellipses represent 95% confidence intervals. In both plots, points represent individual specimens used in the analyses. Different lowercase letters indicate statistically significant differences among groups

We observed that antibiotic treatment significantly reduced centroid size (*F*_(2, 336)_ = 31.19, *p* < *0.001*; Fig. 3A). The reduction in centroid size occurred both in individuals antibiotic-treated with depleted microbiota (G04) and in individuals antibiotic-treated with restored microbiota (G12) (G04 − G00: estimate = −0.116, *p* < 0.001; G12 − G00: estimate = −0.138, *p* < 0.001). However, we found no significant difference in centroid size between the two generations treated with antibiotics (G12 − G04: estimate = −0.022, *p* = 0.067).

**Fig. 4:**
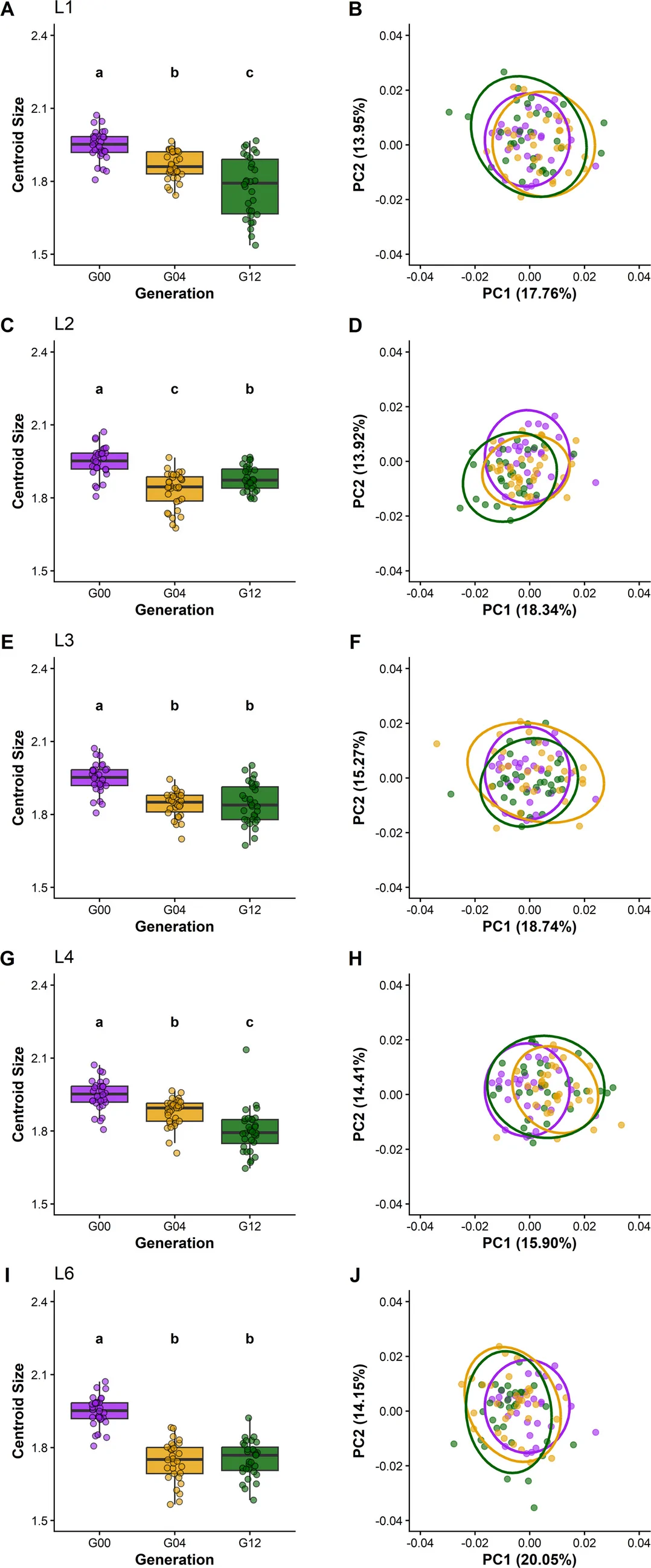
Variation in centroid size and wing shape across generations in five sublines of *Drosophila prosaltans* (L1, L2, L3, L4, L6). All panels display the control generation (G00) in purple, the antibiotic-treated generation with depleted microbiota (G04) in yellow, and the antibiotic-treated generation with restored microbiota (G12) in green. The first column (**A**, **C**, **E**, **G**, **I**) presents boxplots of centroid size for each subline, showing the interquartile range (25%–75%) and median. The second column (**B**, **D**, **F**, **H**, **J**) shows principal component analysis (PCA) plots, represented by the first two components (PC1 and PC2), illustrating wing shape variation across generations. PCA plots were generated from allometry-corrected shape residuals when allometry was significant (L1, L3, L4, L6) and from raw shape data when allometry was not significant (L2). Ellipses represent 95% confidence intervals. Percentages indicate the proportion of shape variation explained by each component. In all plots, points represent individual specimens used in the analyses. Different lowercase letters indicate statistically significant differences among groups

In all sublines, both the generation antibiotic-treated with depleted microbiota (G04) and the generation with restored microbiota (G12) had a smaller centroid size than the control (G00) (Fig. 4A, C, E, G, I). Comparisons between generations with depleted microbiota (G04) and with restored microbiota (G12) revealed variation between sublines: in L1 and L4, the generation with restored microbiota had an even smaller size than the one with depleted microbiota (estimate = −0.090, *p* < 0.001 and estimate = −0.079, *p* < 0.001, respectively); in L2, the opposite occurred, with the generation with depleted microbiota showing a smaller size than the generation with restored microbiota (estimate = 0.048, *p* = 0.011); in L3 and L6, no differences were observed between the two generations (estimate = 0.004, *p* = 0.976 and estimate = 0.008, *p* = 0.9, respectively).

### Wing Shape Variation

The analysis of shape variation across generations, independent of line, revealed a statistically significant allometric effect (3.90%, *p* < 0.0001). Principal component analysis (PCA) indicated that the first two principal components collectively accounted for 33.9% of the total shape variation, with PC1 explaining 18.94% and PC2 explaining 14.96% (Fig. 3B). A multivariate analysis of variance (MANOVA) was performed on the first 17 principal components, which captured over 95% of the wing shape variation.

The results demonstrated that antibiotic treatment induced significant morphological divergence among generations (*F* = 4.55; *Z* = 4.92; *p* = 0.001). Post hoc pairwise comparisons confirmed significant differences in wing shape between all generation pairs (G00 vs. G04: *p* = 0.004; G00 vs. G12: *p* = 0.001; G04 vs. G12: *p* = 0.001). This consistent differentiation across all groups suggests that the effects of antibiotic exposure cannot be disentangled from those of microbiota removal. Moreover, the significant shape difference between the control group (G00) and the antibiotic-treated group with microbiota restored (G12) indicates that the morphological changes persist even after microbiota reconstitution.

Geometric morphometric analysis of the five sublines (L1, L2, L3, L4, and L6) revealed distinct patterns of allometry and wing shape variation across generations. For each subline, we performed a principal component analysis (PCA) followed by a MANOVA based on the first 17 principal components of wing shape, which collectively accounted for over 95% of the total variation. As allometry was significant in sublines L1, L3, L4, and L6, the PCA for these was conducted using the regression residuals of shape on size. In contrast, the covariance matrix was used for subline L2, where allometry was not a significant factor.

The analysis revealed distinct effects of antibiotic treatment and microbiota restoration across the sublines (Fig. 4B, D, F, H, J). While the microbiota depleted generation (G04) exhibited a significantly different wing shape compared to the control in all sublines, the response to microbiota restoration was line-dependent. In L1, wing shape differed significantly between the control (G00) and the microbiota depleted generations (G04; *p* = 0.009), as well as between the microbiota depleted (G04) and restored generations (G12; *p* = 0.019). However, microbiota restoration resulted in a return to the control morphology, with no significant difference between the restored group (G12) and the control (G00; *p* = 0.126). This pattern indicates that recolonization produced an additional shape shift relative to G04 before restoring the control phenotype. In contrast, sublines L2, L3, and L4 showed significant differences among all generational pairs (*p* < 0.01), indicating persistent morphological changes that were not resolved by microbiota restoration. A third pattern was observed in L6, where a significant shape difference was found only between the control (G00) and microbiota-depleted groups (G04; *p* = 0.029), demonstrating that the initial alteration was fully reversed after recolonization, since no significant difference was detected between G04 and G12 (*p* = 0.123). This pattern indicates a direct recovery toward the control morphology without an intermediate shift, as happened in L1.

## Discussion

In this study, we evaluated the effects of tetracycline treatment, used to eliminate *Wolbachia*, on the wing size and shape of *D. prosaltans*. Our results showed a significant reduction in size across all treated sublines and generations. This reduction compared to the control indicates that the antibiotic treatment affected the wing size, and that this effect was not reverted by microbiota restoration. Regarding wing shape, all sublines showed modification in the generation antibiotic-treated with depleted microbiota (G04), and after recovery, two sublines returned to the initial shape of the control. These patterns in wing shape are consistent with effects associated with microbiota disruption and antibiotic exposure. However, even starting from the same genetic profile, the recovery was not sufficient to restore the original shape in three of the five sublines. This suggests that the antibiotic-induced disruption had persistent effects on wing morphology and that the recovery process may have resulted in a microbiota different from the original one.

The sustained size reduction in all sublines, observed even after microbiota restoration, suggests a lasting impact of the antibiotic treatment. This persistent reduction may be linked to tetracycline-induced gut morphology changes, which can impair nutrient absorption efficiency (Zhang et al. 2023). Furthermore, antibiotic exposure can alter the expression of genes associated with essential metabolic, digestive, and immune pathways, with effects that may be transmitted to subsequent generations (Zhang et al. 2023; Kriete and Scott 2025). Together, these mechanisms suggest that tetracycline exposure may induce long-term biological effects, potentially involving epigenetic regulatory changes or lasting alterations in developmental trajectories. Prolonged antibiotic exposure may disrupt metabolic and regulatory pathways in ways that generate stable phenotypic shifts even after treatment cessation (Zhang et al. 2023; Kriete and Scott 2025). While the present study does not directly test these mechanisms, they provide plausible explanations for the sustained morphological patterns observed. Additionally, one treated subline (L5) failed to persist beyond the fifth generation. Although the specific causes cannot be determined, this pattern is consistent with a potential fitness cost associated with antibiotic exposure, further suggesting that tetracycline treatment may impose lasting biological constraints on treated individuals.

Regarding wing shape, all sublines exhibited modifications compared to the control in the generation antibiotic-treated with depleted microbiota (G04). We expected a return to the initial control shape after microbiota restoration, but this occurred in only two of the five sublines. The observed morphological differences suggest that the antibiotic may have exerted negative effects on individual development (Shabbir et al. 2024). The elimination of *Wolbachia* also removes other bacteria present in the organism, such as the intestinal microbiota (Schneider et al. 2019; Weiland et al. 2022), which can lead to deficiencies in nutrient absorption and production (Kriete and Scott 2025). Furthermore, recolonization after antibiotic treatment may result in a microbiota that differs in diversity from the original (Weiland et al. 2022). Although the present study did not directly assess microbiota community variation, such changes in composition could represent an additional mechanism contributing to the observed patterns. Therefore, microbiota disruption associated with antibiotic treatment may have contributed to the shape differences observed in the generation antibiotic-treated with depleted microbiota. Following recolonization, the microbiota of the antibiotic-treated generation with restored microbiota (G12) may have differed in composition from that of the control generation (G00). This difference could also have contributed to the persistent shape divergence observed in some sublines. Even though the sublines shared the same genetic background, variations in the expression of genes associated with essential metabolic pathways may have influenced the observed differences in wing shape, which might or might not persist after the antibiotic exposure ended (Zhang et al. 2023; Kriete and Scott 2025).

The effects of tetracycline on size reduction and wing morphology observed in the sublines may have important implications for their use in experiments. In drosophilids, females tend to choose larger males for copulation, using size as a proxy for reproductive quality (Partridge and Farquhar 1983; Partridge et al. 1987; Lev and Pischedda 2023). Furthermore, during copulation, males produce sounds by vibrating their wings as a component of courtship (Spieth 1974), a behavior fundamental for successful mating in *D. prosaltans* (Colyott 2019). Variations in both wing size and shape can alter the sound patterns produced, thereby influencing female mate choice (Menezes et al. 2013). Therefore, treatment-induced alterations in wing size and shape could potentially influence courtship performance in treated males, suggesting interesting directions for future mate choice experiments. To evaluate this influence, preference tests should compare untreated males with treated males that have undergone gut microbiota recovery but display different wing morphology.

Studies that evaluate the relationship between *Wolbachia* and its hosts using antibiotic treatments tend to attribute the observed results solely to the removal of the symbiont. However, even in studies where the host is not infected by *Wolbachia*, antibiotic treatment can generate effects such as modifications in wing asymmetry in *Drosophila*, alterations in gene expression patterns in flies, and impairments in food digestion in ticks (Büyükgüzel et al. 2020; Zhang et al. 2023; Kriete and Scott 2025). In contrast to most studies that evaluate a single treated line, this research examined sublines sharing the same genetic background and revealed distinct patterns of effect manifestation. Consequently, our results highlight the importance of considering the effects of antibiotic treatment beyond the removal of *Wolbachia*. It is important to note that only males were evaluated in this study, and the effects of tetracycline treatment used to remove *Wolbachia* may differ between sexes. Therefore, the morphological patterns observed here cannot be directly generalized to females or to the species as a whole. Further studies encompassing both sexes are needed to clarify whether these findings are sex-specific or applicable to the species as a whole.

Importantly, the observed phenotypic patterns likely reflect multiple, non-mutually exclusive mechanisms. Effects may arise from *Wolbachia* removal per se, as endosymbionts are known to influence host metabolism, nutrient allocation, and developmental stability (Burdina and Gruntenko, 2022; Strunov et al. 2022; Karpova et al. 2023; Lindsey et al. 2025). Alternatively, or concurrently, changes may result from direct or indirect effects of tetracycline exposure, including mitochondrial dysfunction, altered gene expression, and long-term metabolic disruption (Ballard and Melvin 2007; Houtkooper et al. 2013; Zhang et al. 2023; Kriete and Scott 2025). Because antibiotic treatment simultaneously eliminates *Wolbachia* and perturbs host cellular and microbial processes, disentangling these pathways remains inherently challenging. Although microbiota restoration was performed to minimize the effects of microorganism removal, it is still not possible to fully separate the effects of *Wolbachia* elimination from those of antibiotic exposure. A design explicitly aimed at isolating these components would require multiple independent treated and untreated line, which, while informative, would reduce the advantage of our experimental approach that maintained a shared genetic background across sublines. The loss of the L5 subline during the recovery period further supports the possibility that tetracycline exposure combined with microbiota disruption may impose substantial biological costs on treated individuals. Therefore, the persistent morphological changes observed here are more plausibly explained by the combined effects of symbiont removal and antibiotic treatment than by either factor acting independently.

Our findings show that tetracycline treatment reduced *Wolbachia* to PCR-undetectable levels and was associated with persistent phenotypic alterations in *D. prosaltans* that remain even after microbiota restoration. Considering the central role of wings in courtship and acoustic signaling, such changes may have important consequences for behavioral outcomes, including mate choice (Colyott 2019). Antibiotics are increasingly prevalent in natural environments, raising the possibility that organisms may also experience long-term phenotypic effects under field conditions. Collectively, our results emphasize a critical point: phenotypic differences observed following antibiotic treatment may not solely reflect microbiota or symbiont removal, but also the lasting effects of antibiotic exposure itself. Consequently, interpretations in host–symbiont studies should be made with caution, with explicit consideration of potential antibiotic-induced effects on host morphology, physiology, and behavior.

## Supplementary Information

Below is the link to the electronic supplementary material.

ESM 1 (DOCX 18.2 KB)

ESM 2 (DOCX 15.0 KB)

## Data Availability

The datasets and R code generated for size and shape analyses during the current study are available in the Zenodo repository at https://doi.org/10.5281/zenodo.22941126.
