# Supplementary material for: Antibiotic Treatment Reduces Wing Size and Alters Wing Shape in a Neotropical Drosophila (Diptera: Drosophilidae)
Source: Neotrop Entomol. 2026 Oct 29;55(1):99. doi: 10.1007/s13744-026-01442-2 (PMC13719771; doi:10.1007/s13744-026-01442-2)
Supplement: Supplementary file 1 [file 13744_2026_1442_MOESM1_ESM.docx]

**Supporting Information 1**

**Banana-Barley Culture Medium**

Materials Required

- Corn syrup - 9.5 g
- Banana - 13.8 g
- Distilled Water - 100 ml
- Beer Yeast - 2.8 g
- Barley Flour - 3.0 g
- Agar-agar - 0.8 g
- Methylparaben 0.22 g
- 100 % Ethanol

Procedures

Prepare this medium the day before you plan to use it. Keep it at room temperature. If it´s too wet, flies may sick to it and die.

1. In an Erlenmeyer, place corn syrup, banana, and 50 ml of water.
2. Cook the mixture in a microwave until getting a homogeneous mixture.
3. Add beer yeast, barley flour, agar-agar and the remaining water.
4. Cook long enough to homogenize the ingredients and dissolve the agar.
5. Cool the medium until it is comfortable to the touch.
6. In a separate container, dissolve methylparaben in a small amount of ethanol, then add this to your mixture.
7. Pour the medium into sterilized containers Let it solidify at room temperature.
8. Before using, sprinkle granulated biological yeast on top of the medium.
