# Supplementary material for: Antibiotic Treatment Reduces Wing Size and Alters Wing Shape in a Neotropical Drosophila (Diptera: Drosophilidae)
Source: Neotrop Entomol. 2026 Oct 29;55(1):99. doi: 10.1007/s13744-026-01442-2 (PMC13719771; doi:10.1007/s13744-026-01442-2)
Supplement: Supplementary file 2 [file 13744_2026_1442_MOESM2_ESM.docx]

**Supporting Information 2**

|  | **Generation** | | |
| --- | --- | --- | --- |
| **Line** | G0 | G04 | G12 |
| **Control** | 30 | - | - |
| **Subline L1** | - | 30 | 30 |
| **Subline L2** | - | 32 | 30 |
| **Subline L3** | - | 30 | 33 |
| **Subline L4** | - | 31 | 32 |
| **Subline L6** | - | 30 | 31 |

**Supporting Table S2.1** Number of individuals used in the morphometric analyses for each line and generation evaluated. The control generation (G0) corresponds to the individuals from the Pro 101 line without tetracycline treatment. Generation G04 corresponds to individuals antibiotic-treated with depleted microbiota. Generation G12 corresponds to individuals antibiotic-treated with restored microbiota.
